# Organizations’ Strategies to Improve Implementation of Universal Accessibility Principles: Protocol for a Scoping Review

**DOI:** 10.2196/33641

**Published:** 2022-07-11

**Authors:** Maëlle Corcuff, François Routhier, Emmanuelle Paquette-Raynard, Martine Gagnon, Alfiya Battalova, Claudel Mwaka, Marie-Eve Lamontagne

**Affiliations:** 1 Département de réadaptation Université Laval Québec, QC Canada; 2 Université Laval Québec, QC Canada; 3 University of British Columbia Vancouver, BC Canada

**Keywords:** universal accessibility, universal design, public organizations, implementation strategies, disability, person with disability, human rights, thematic analysis, occupational health, workplace

## Abstract

**Background:**

Since the publication of the Convention on the Rights of Persons with Disabilities, several countries have adopted laws, policies, and action plans to improve the universal accessibility of environments to improve inclusion and social participation of all citizens. Different organizations are involved in the application of these measures.

**Objective:**

The aim of this study is to identify strategies that are contextually appropriate and provide guidelines for organizations to promote successful implementation of universal accessibility.

**Methods:**

We will conduct a scoping review identifying implementation strategies of universal accessibility measures in local organizations using the Arksey and O’Malley framework. We will search in Medline, CINHAL, Urban Studies Abstracts, ABI Inform, and Social Sciences Full Text from 2006 until today, following the adoption of the Convention on the Rights of Persons with Disabilities. Two reviewers will independently select studies for inclusion and will extract the data. A descriptive and thematic analysis of the characteristics of the identified implementation strategies will be performed.

**Results:**

Implementation strategies will be summarized in tables. They will then be linked to various constructs and domains listed in the Theoretical Domain Framework to identify barriers and facilitators of organizations’ uptake of evidence-based strategies of implementation.

**Conclusions:**

We will tabulate the characteristics of the included studies and the outcomes of implementation strategies in them. The results of this scoping review are expected to help the research community in various fields, local organizations, and stakeholders to identify better ways to improve implementation strategies of universal accessibility practices.

**International Registered Report Identifier (IRRID):**

PRR1-10.2196/33641

## Introduction

### Background

Universal accessibility is defined as the character of an environmental design that allows all individuals to carry out their activities independently, with equity and in an inclusive approach [1]. It aims to create better and adapted environments and to provide equal access to buildings, services, resources, and activities for the population [2,3]. The Convention on the Rights of Persons with Disabilities (CRPD), adopted in 2006 by the United Nations (UN), put forward the importance of this concept [4]. The general objective of this convention is to improve the life of people with disabilities and to promote a significant change in social development in several spheres of life (health and mental health, accessibility, independence and autonomy, education, employment, housing, etc) to facilitate the inclusion of these people [4-7]. The recognition of the importance of universal accessibility is even more relevant today. Indeed, the global social context of modern societies, demographic changes, and the aging population are leading to a greater proportion of people with disabilities living in urban areas [8]. Since the adoption of the CRPD, several countries have stepped in to create more accessible environments for all individuals [9]. Universal accessibility is an important catalyst for the social and economic participation of every individual. It touches several aspects of citizens’ lives, such as quality of life, sense of belonging, health, social inclusion and participation, and employment [9-11]. For this scoping review, the concept of universal accessibility includes a built environment. Accessibility for all to build an environment helps to reduce risks of social exclusion by providing opportunities for activities and reducing isolation and loneliness [12,13]. It helps to promote social participation by facilitating access to employment, decision-making, and various activities. These benefits also contribute to meet the important sociopolitical objective to improve the quality of life of communities [10].

Although the CRPD is an initiative of the UN grouping of countries, in many cases, local jurisdictions are more involved in implementing the various recommendations, since a built environment is generally the responsibility of local governments. For example, Canada, a member of the UN, responds to the CRPD by creating the Accessible Canada Act [14] as the province of Quebec proposes the act to secure handicapped persons in the exercise of their rights with a view to achieving social, school, and workplace integration [15]. The creation of accessible environments is regulated in the various action plans established by local governments that are themselves governed by the provincial law. Thus, municipalities are directly concerned and responsible for the implementation of measures that will allow the creation of accessible environments. The United States created the American with Disabilities Act [16], which prohibits discrimination against people with disabilities in several areas. Each state must then individually set up their laws so local governments can implement policies that propose measures to create accessible environments. In the literature, the involvement of local governments in conventions about international issues (climate change, racial discrimination, cultural heritage, tobacco control, child welfare, etc) is demonstrated. From these conventions, provinces and municipal governments develop their own legislation, as seen in Canada. The literature also shows that municipalities have a key role to play in addressing the social and global issues, given their proximity to citizens [17-19]. For example, the UNESCO (United Nations Educational, Scientific and Cultural Organization) 2003 convention about cultural heritage highlights the role of local governments worldwide to actively participate in the safeguarding and the promotion of their culture [20]. Pineda et al [19] also argue that “addressing accessibility will require assessing and addressing gaps in infrastructure management, municipal codes land use, transportation planning, housing and community development, mobility, social services, and broader monitoring of human rights at the local level.”

### Prior Work

While the implementation of universal accessibility measures, as the design of environments and services usable for all people [4], has an important impact on the social participation and quality of life of people living with disabilities [2,9,21-23], it also benefits all populations, regardless of disability, by facilitating access to environments for all [2,24,25]. An accessible environment allows everyone, with or without disability, to use it equitably and provides added value to the entire population, such as the elderly experiencing loss of mobility, parents with children in strollers, tourists with their suitcases, etc. Universal accessibility is important in many areas of the citizens’ lives, such as public services, educational institutions, housing policies, leisure, health care, cultural, social, and political participation, transportation, information, built environment, territory development, and others [10]. However, this scoping review will focus on the built environment involving the local government, as several stakeholders are consequently involved in the implementation such as governments, municipalities, community-based organizations, or researchers. It is especially common for local governments to adopt a set of measures to follow the guidelines for accessible design of the physical environment [26,27], making the implementation of universal accessibility principles a purview of employees of these organizations.

The implementation of universal accessibility principles is, however, a complex initiative [28]. While implementation is often perceived as linear and easy, implementation scientists suggest that implementation strategies should be tailored to a specific context and be carefully designed to allow the actualization of the innovation, such as universal accessibility principles [29-31]. It is also well established that the implementation of a given innovation is influenced simultaneously by various determinants [31,32]. The level of knowledge, beliefs of the actors involved, or the availability of resources are some examples of determinants influencing implementation strategies and behaviors [33]. Knowing what determinants influence the implementation of principles can help guide decisions about what strategies to adopt on the basis of how well they address the key determinants that can facilitate the implementation of universal accessibility measures [34]. However, little is known about how local governments have adopted implementation strategies to address the various determinants and environmental barriers in the application of universal accessibility principles. Otherwise, we do not know much about municipal perspectives or evaluation toward accessibility practices, or about the nature, targets, and effectiveness of the various strategies chosen by organizations to improve the implementation of universal accessibility principles. Such knowledge is crucial to a better implementation and use of universal accessibility principles in organizations, including, but not limited to, municipalities. Thus, the objectives are to describe the strategies that are used by municipalities to implement better universal accessibility measures.

### Identifying the Research Question

To identify the appropriate research question, the authors, specialized in the accessibility field or in implementation sciences (MC, MEL, and FR), consulted each other to identify relevant questions that address a gap in scientific and organizational knowledge. This scoping review aims to answer the research question, “What are the implementation strategies used by public organizations to apply universal accessibility measure?” The identification of the question is based on gaps in the literature specifically related to implementation strategies in local organizations, the effectiveness of strategies in implementing universal accessibility measures, and to address the needs of organizations to better document these strategies for improvement of universal accessibility. This scoping review will focus on implementation strategies used only by the local government and municipalities. This choice is justified by the fact that they are directly concerned within the implementation of universal accessibility measures, not only because of their duty and legal responsibility to create laws and policies in this sense, but also because they have the direct and proximal impact on the creation of infrastructures, etc, on the daily life of citizens.

### Aim of This Study

The aim of the proposed study is to explore the implementation strategies used by local governments to implement universal accessibility measures. The specific objectives are to (1) identify the different strategies implemented by local governments in relation to the principles of universal accessibility, (2) understand how effective these strategies are, and (3) identify the facilitators and barriers to the implementation of the principles of universal accessibility.

## Methods

### Overview

This knowledge synthesis will take the form of a scoping review. This type of knowledge synthesis is adapted to the needs of the subject since it will allow us to (1) examine the extent, range, and nature of research activity; (2) determine the value for undertaking a full systematic review; (3) summarize and disseminate research findings; and (4) identify research gaps in the existing literature [35-37]. This seems appropriate given the innovative character of the research topic and the limited knowledge available, as it will help to determine the scope of the literature on the topic and provide a clear indication of the volume of literature and studies available [1]. We will use the framework described by Arksey and O'Malley [36], which involves 5 different stages: (1) identifying the research question, (2) identifying relevant studies, (3) selecting studies, (4) charting the data, and (5) collating, summarizing, and reporting the results. Step 1 is described in the *Introduction*, while step 5 is described in the *Results*. The method will be reported in accordance with the PRISMA (Preferred Reporting Items for Systematic Reviews and Meta-Analyses) extension guidelines for scoping reviews [38].

### Identifying Relevant Studies

A consultation with research librarians (EPR and MG) specialized in databases relevant to both fields—environment and implementation—is supporting the definition of the parameters of the strategy. For the purposes of this scoping review, academic and gray literature will be consulted to identify all relevant information about the subject. As the creation of accessible environments is addressed in several disciplines and fields of research (social sciences, medicine and rehabilitation, psychology, public health, geography, urban studies, environment, and engineering), interdisciplinary electronic databases will be consulted: Medline (EBSCO), Urban studies abstract (EBSCO), CINHAL plus full text (EBSCO), ABI Inform (Proquest), and Social Sciences Full Text (EBSCO). Telecommunications-related studies will be excluded since the scoping review subject is about the built environments. All empirical study designs will be considered in this scoping review, including quantitative and qualitative methods and mixed methods studies. Gray literature sources such as local organizations’ or municipalities’ reports in local governments’ websites or those in Google Scholar will also be searched with the key words “universal design” or “universal accessibility.” Through controlled vocabulary, various keywords and related terms will be included: (1) “Universal access*” OR “architectural design” OR “universal design,” (2) “Local govern*” OR “Municipalit*.” The following is an example of the search strategy used in Medline: ((Universal access* OR “universal design” OR “design for all”) OR (MH “Universal Design+”) OR (MH “Facility Design and Construction+”)) AND ((“Local govern*” OR City OR Cities OR municipal*) OR ((MH “Cities”) OR (MH “Local Government”)). Articles will have to be published since 2006 to the day of this writing; that is, at the time of the establishment of the UN CRPD. Search results will be imported into the Covidence platform, where the duplicates will be automatically removed.

### Study Selection

To be included, the studies will need to adhere to the following inclusion criteria: (1) having been published after 2006, (2) be an empirical study (qualitative, quantitative, or mixed methods), (3) be relevant to local governments or municipalities, and (4) be written in French or English. The exclusion criteria are as follows: (1) telecommunications accessibility studies (websites, media, TV, etc) and (2) studies evaluating accessibility. Studies on the implementation of accessibility of built environments for people with disabilities (including seniors). The study selection will consist of 2 steps: (1) screening of title and abstract and (2) review of the full text will be done on the Covidence platform. At first, 2 doctoral students (MC and CRM) will independently screen the titles and abstracts. Conflicts will be resolved through discussion and a third reviewer (AB) will be involved if a consensus cannot be reached. In the second selection step, all full-text versions of the articles will be obtained and read. MC and CRM will decide independently of the final inclusion of the article in the review. The reasons of exclusion for each article will be noted. Occurring disagreements will be discussed with a third reviewer (AB) and consensus will be reached.

### Charting the Data

The selected articles and the Endnote references with attached files will be imported in the NVivo software. Sources will be classified using attributes such as author name or publication date. Each article will be read, annotated, and coded with emerging themes. Because of its relevance in the implementation field, the coding tree will be inspired by the one used in the implementation review study of Aregbesola [39] (see Textbox 1) and will be composed of 2 main codes: the study details and the intervention described. The subcodes will be broken down as follows: the study details will include the name of the first author, year of publication, country, study design, study period, study objective (what is being implemented), and area of study. The use of any of the implementation strategies, dissemination strategies (developing messages and materials and distribution of evidence-based information), implementation process strategies, integration strategies, capacity building, and the numbers and types of implementation strategies used will be extracted in the intervention code. Textbox 1 shows the extraction chart categories, which will be imported in NVivo software. A pilot of this coding tree will be carried out on 5 studies to determine whether other themes are emerging. A word frequency query or a matrix coding query could be used to determine which themes are most and least represented within the implementation strategies and to verify the accuracy of the information extracted.

**Coding tree for analysis of implementation strategies.**

Study details:

- First author
- Year of publication
- Country
- Study period
- Study objective (what is being implemented)
- Area of study

Interventions, policies, or strategies:

- Use of any of the implementation strategies
- Dissemination strategies (messages, materials, distribution, or evidence-based interventions)
- Implementation process strategies
- Integration strategies

## Results

The results of the studies will be reported in tables. Specific characteristics and outcome measures of included studies will be presented in a table. Each type of implementation strategy will be summarized, along with the study designs and the effects produced, in the table. The strategies used by the organizations will be linked to various constructs and domains listed in the Theoretical Domain Framework [40]. This implementation sciences framework will allow us to identify barriers and facilitators of organizations’ uptake of evidence-based strategies of implementation [41]. The impact of each construct on the implementation of universal accessibility measures will be described in the table form. These different steps will allow us to answer the research question by identifying the implementation strategies used by organizations to improve the universal accessibility of environments. A participatory process is planned during the analysis of the scoping review by soliciting representatives of local organizations to ensure that the scoping review meets the needs of municipalities and citizens. Three meetings with the research team and the representatives of local organizations will be planned. They will be held in person or on Zoom (Zoom Video Communications), depending on the state of the pandemic at the time. Representatives of local organizations will be contacted initially to present them the project, the objectives, and the analysis process that will be conducted. The first 10 articles will be analyzed, and the second meeting will be used to discuss this preanalysis and adjust it as needed, based on comments and suggestions from the representatives. Once all parties have agreed on the analysis process, an analysis of all articles will be conducted. Finally, a last meeting will be scheduled to validate the results of the analysis and ensure that they meet the needs of local organizations. We expect the scoping review to be completed by December 2022.

## Discussion

### Principal Findings

In summary, this scoping review will empirically identify the nature, scope, and effectiveness of implementation strategies in municipal settings to improve environmental accessibility. In a world with an increasing aging and a disabled population, it is essential to address the issue of accessible environments for all [42-45]. Public organizations, from a local to national level, have an essential role to play in improving universal accessibility services and practices and in developing better implementation strategies [27,46].

### Comparison to Prior Work

To the best of our knowledge, little research has documented the effectiveness and nature of implementation strategies used specifically by public organizations in creating accessible environments [9,12,34,47,48]. Therefore, this scoping review will provide evidence-based information on the different implementation strategies that have been used to date in the field of universal accessibility by public organizations and will characterize domains influencing the implementation of such an innovation. It will also contribute to literature and research advancement by identifying implementation strategies that could possibly be applicable in other contexts. It will help reduce the gap between knowledge owners and knowledge users for the implementation of universal accessibility measures [9]. This will potentially influence future research in the social sciences, urban and environmental studies, rehabilitation, or public health research by providing new knowledge in implementation science and in universal accessibility within organizations.

This scoping review could also influence the important need of better coordination in practices and policies in regard to the implementation of accessibility practices within organizations [49]. The use of relevant implementation strategies by organizations could bring the stakeholders to improve their capacity to establish better initiatives and strategies of the implementation of universal accessibility principles [34]. It will likely help them overcome barriers to the implementation of such measures. Organizational practices toward universal accessibility are crucial to the elaboration of quality interventions and to improve the accessibility of environments and the quality of life of people living with disabilities as well as those of their cocitizens without disabilities. This will help increase social participation and inclusion of people living with disabilities [10]. It may also impact economic, social, and overall health dimensions of populations [50]. Better accessibility to services and infrastructures could ultimately reduce exclusion and various risk factors associated with health and lack of employment and will improve general mobility for the entire population [12,13,51]. It will allow every citizen to participate fully in different aspects of society.

### Future Directions

Conducting a scoping review of the knowledge available across many fields will assist all city employees, whether they are managers, civil servants, professionals, blue collar, or seasonal employees, to develop better implementation strategies of universal accessibility measures [51]. It will also be beneficial for publishers, policy makers, researchers, teachers, and students to be more informed on the various component of appropriate practices in the field of implementation strategies toward universal accessibility practices [8].

### Dissemination Plan

To share the knowledge gained from this scoping review, a dissemination plan has been developed. The results of the scoping review will be published in a scientific paper by December 2022. They will also be presented at a provincial and an international conference. Finally, the results will be communicated to the City of Quebec, to pursue the objectives of the partnership, to find innovative solutions in knowledge mobilization, and to facilitate the implementation of universal accessibility measures within the municipality.

### Conclusions

The fact that the research question for this scoping review was refined through rigorous questioning by specialized researchers and that a participatory process involving local organizations representatives is planned to analyze and report the results, demonstrates the concern to respond to a real need felt by local organizations and the research community in the field of universal accessibility. The complex and challenging aspects of implementation science, particularly of the implementation of universal accessibility measures and its success depending on the effectiveness of the strategies used and the various domains, justify the relevance of conducting this scoping review. The identification of robust implementation strategies, processes, and outcomes is essential to the creation of accessible environments that enable inclusion and social participation for all, regardless of disability. Given the growing need of disabled populations and the importance of the sociopolitical objective to improve the quality of life of communities, addressing ways to reduce environmental barriers, better implementation strategies initiatives within public organizations will contribute to future practices in this field.

## Abbreviations

CRPD: Convention on the Rights of Persons with Disabilities
UN: United Nations
UNESCO: United Nations Educational, Scientific and Cultural Organization
